# Quantitative Insight into PCA Formation following Different Chlorhexidine Activation Methods in Endodontic Treatment

**DOI:** 10.3390/molecules28166159

**Published:** 2023-08-21

**Authors:** Barbara Czopik, Aneta Woźniakiewicz, Natalia Świętoniowska, Joanna Zarzecka, Michał Woźniakiewicz

**Affiliations:** 1 Department of Conservative Dentistry with Endodontics, Faculty of Medicine, Institute of Stomatology, Jagiellonian University Medical College, Montelupich 4 St., 31-155 Kraków, Poland; j.zarzecka@uj.edu.pl; 2 Department of Analytical Chemistry, Faculty of Chemistry, Jagiellonian University, Gronostajowa 2, 30-387 Kraków, Poland; aneta.wozniakiewicz@uj.edu.pl (A.W.); michal.wozniakiewicz@uj.edu.pl (M.W.)

**Keywords:** chlorhexidine, PCA, endodontic treatment, root canal treatment, endodontics, root canal irrigation

## Abstract

The aim of the study was a quantitative analysis of p-chloroaniline (PCA) formation during 2% CHX activation with US and MDI methods in a root canal-like environment with the HPLC-DAD method and, thus, a safety assessment of US and MDI agitation of CHX in endodontic treatment. Two percent CHX was activated with the US method using ISO 30 and 35 K-file, and the MDI method using ISO 30.06 and 35.06 GP cones for 15, 30, 60, and 90 s. PCA concentration was assessed with the HPLC-DAD method. PCA concentration was also assessed for 2% CHX after 0, 3, 18, and 21 days of storage in ambient conditions. PCA was detected in all samples in all methods of activation. The concentration of PCA was dependent on time of activation in US ISO 30 and ISO 35 group (*p* < 0.05). In the MDI ISO 30.06 and ISO 35.06 groups, a similar trend was observed but without statistical significance (*p* > 0.05). PCA was detected in shelf-stored 2% CHX and the concentration was related to the time of storage. PCA is released after CHX activation with US and MDI, but mean concentrations are not higher than those observed from self-degradation of shelf-stored 2% CHX.

## 1. Introduction

The main objective of root canal treatment (RCT) is to prevent or cure apical periodontitis (AP) by achieving effective three-dimensional chemo-mechanical preparation of endodontic space. Cleaning of root canal system is performed by mechanical preparation with hand or rotary instruments and by chemical preparation with endodontic solutions. Traditional shaping objectives, now redefined from “shaping and cleaning” to “shaping for cleaning”, emphasize the role of chemical preparation as the only part of the treatment that can disinfect and debride all elements of the root canal system, including isthmi, apical delta, lateral canals, and dentinal canaliculi [1,2].

The most important chemical solution used in endodontics is sodium hypochlorite (NaOCl) [3]. It is used in concentrations of 2–5.25% due to its excellent tissue dissolving capacities, broad antimicrobial spectrum, and ability to remove the organic component of the smear layer [4]. NaOCl is used throughout the whole treatment in order to eradicate microbiota, chemically prepare parts of the endodontic space that cannot be reached with mechanical preparation, and in the final irrigation protocol for additional antimicrobial effect and removal of the organic part of the smear layer [5].

Chelators-40% citric acid (CA) or 17% ethylenediamine tetraacetic acid (EDTA) have the ability to bind calcium ions, demineralize and soften the dentin, and increase its permeability [6], and are, therefore, used in the final irrigation protocol to remove the inorganic component of the smear layer [7,8].

Two percent chlorhexidine (CHX) is the third most widely applied solution in endodontics. It is used for additional antibacterial action due to its broad antimicrobial spectrum and clinical phenomenon called “substantivity” [9]. Chlorhexidine is dedicated as a last flush in the final irrigation protocol to enhance the disinfection of the endodontic space and preserve a prolonged antimicrobial effect of the chemical preparation [9].

The mode of irrigants application into endodontic space is another factor influencing the effectiveness of the root canal system disinfection [10]. The conventional way of irrigant delivery: syringe and needle irrigation (SNI) fails to fulfill the criteria of effective chemical preparation, which is to distribute the irrigant throughout places that cannot be reached with mechanical preparation [10]. Therefore, irrigants agitation techniques (IAT) began to be used as dental practitioners became more aware of the subject of endodontic hydrodynamics [11]. IAT methods include passive ultrasonic irrigation (PUI), when irrigants are activated with ultrasounds (US), sonic activation (SA), as well as manual dynamic irrigation (MDI). All those methods aim at changing the flow of root canal irrigants from laminar into turbulent, which increases the action of chemical solutions distributed throughout the endodontic space by ultrasonically, sonically, or manually generated streaming forces [12]. This results in more effective disinfection of the root canal space [12,13].

PUI, the method using US for activation, is believed to be the most effective way for enhancing chemical preparation in the aspect of debridement of root canals [12,13,14], for removal of microbiota from the endodontic space [15,16], and for better penetration of solutions into dentinal tubules [15,17]. The intensity of ultrasonic activation influences the energy transmission by a freely oscillating file inside the root canal space filled with irrigating solution [13,17]. This results in acoustic streaming not only at the tip but all around the ultrasonic file [13,14,17]. PUI is considered a golden standard among methods of irrigant activation and is most preferred by endodontic practitioners.

MDI improves antimicrobial action of endodontic irrigants, enhances debridement of the endodontic space, and provides apical patency [18]. In this technique, gutta-percha (GP) cone is used with up and down motion in the root canal with the frequency of at least 100 push/pull movements per minute [18]. MDI can be applied for all irrigants, does not require application of advanced devices and specially designed files, and is dedicated for less equipped and less experienced practitioners [18].

As other root canal irrigants, CHX was proposed to be activated with PUI in order to increase its antimicrobial action and penetration into dentinal canaliculi [17]. However, ultrasonic activation increases the temperature of 2% CHX solution [19,20] and heating of CHX was reported to cause the formation of a highly toxic compound para-chloroaniline (PCA) [21]. Therefore, it was hypothesized that CHX activation with the US method leads to PCA release. Nevertheless, direct proof for this potential toxic effect of CHX agitation with either PUI or MDI method has not been provided up to date. Moreover, a quantitative analysis of PCA released from US or any other IAT of 2% CHX has not previously been conducted.

Constant exposition to PCA was proved to cause multiple types of cancer in rats: fibrosarcoma and osteosarcoma in the spleen, pheochromocytoma in the adrenal gland, hemangiosarcoma in the liver, as well as methemoglobinemia in humans [22,23]. Moreover, brown precipitate, which is produced in the reaction of CHX and NaOCl and contains PCA, cannot be removed from the root canal walls completely and can compromise the quality of root canal obturation [22,24], which can result in treatment failure.

The aim of the study was a quantitative analysis of PCA formation during activation of 2% CHX with the US and MDI methods in root canal-like conditions with high-performance liquid chromatography (HPLC) technique with a DAD detector and, thus, the assessment of the safety of ultrasonic and manual agitation of CHX solutions in endodontic treatment. Moreover, we decided to conduct a quantitative analysis of the amount of PCA released from shelf-stored 2% CHX in ambient conditions in order to compare those results with the results obtained in experimental groups (2% CHX activated with the US and MDI method). The null hypothesis was that: (1) there will be PCA release from 2% CHX activated with the US method, as the temperature of the solution will rise, and that the amount of PCA will correlate with the time of activation and will be higher for a thicker file; (2) there will be no PCA release from 2% CHX activated with the MDI method, as there will be no temperature rise, regardless of the size of the GP cone and time of agitation; (3) there will be PCA release from shelf-stored 2% CHX in ambient conditions and the amount of PCA will correlate with the time of storage but will be significantly lower than the amount released in the US experimental group.

## 2. Results

### 2.1. CHX Activation and Temperature Effect

The changes of temperature of 2% CHX solution before and after activation with US and MDI methods are presented in Table 1. US activation caused a noticeable increase of temperature, while in case of MDI, no change of temperature was detected.

### 2.2. PCA Release during US and MDI Activation of 2% CHX

**Figure 1:**
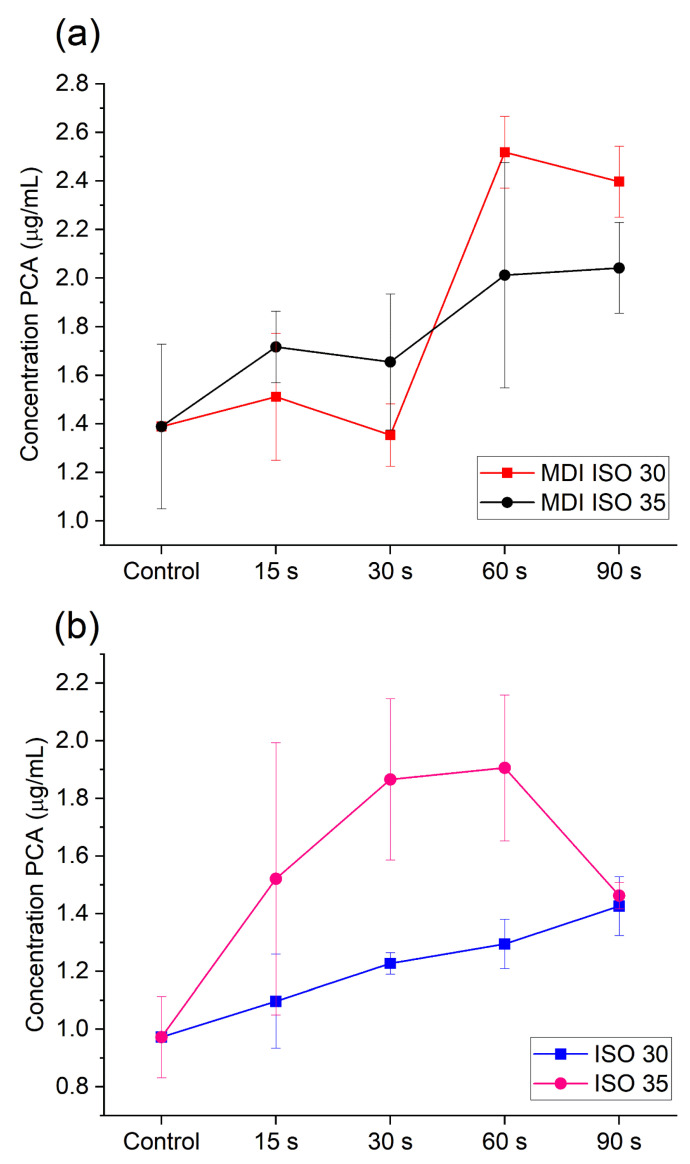
PCA formation upon time of activation of 2% CHX with (**a**) the MDI method and (**b**) the US method.

The chromatographic analysis of activated 2% CHX enabled us to determine PCA release during US activation. For the ISO 30 file, the amount of PCA released increased with the time of activation. When 2% CHX was activated with a thicker file (ISO 35), PCA formation was also observed, but it did not change significantly with time (Figure 1b). Nevertheless, the ANOVA analysis revealed that there is a statistical difference between mean PCA concentration in the case of the ultrasonic activation method with ISO 30 (*p* = 0.02; MS_between_ = 0.116, MS_error_ = 0.014). Particularly, the Tukey post-hoc test revealed that application of US ISO 30 generates statistically significant higher amounts of PCA than those found in the control group if it is applied for 60 s (q_control-60s_ = 0.041, *p* < 0.05) or 90 s (q_control-90s_ = 0.004, *p* < 0.05). The increase in PCA concentration also results in a significant difference between results for 60 and 90 s (q_control-90s_ = 0.036, *p* < 0.05), which supports the observation of positive effect of time on PCA generation. In the case of the US ISO 35 method, the assumption on the equality of variances was not met; thus, the Kruskal–Wallis test was applied. The analysis show that there is a difference between the control group and processed samples (H_4,n=17_ = 12.340, *p* = 0.015); however, the particular difference may be found for 60 s, taking into account the post-hoc Dunn test results (*p* = 0.021).

PCA was also detected in 2% CHX solution after activation with the MDI method. When CHX was agitated with the ISO 30.06 GP cone, a rise of PCA formation was observed for 60 and 90 s time of activation. When the ISO 35.06 GP cone was used, PCA was also released and its amount decreased at the initial stage of activation, which was followed by a rise of its concentration in 60 and 90 s. In this case, the ANOVA analysis revealed that there is a statistical difference between mean PCA concentration released in 60 and 90 s of MDI ISO 30.06 activation (*p* = 0.0002; MS_between_ = 0.928, MS_error_ = 0,049), which was indicated by the results of the Tuckey post-hoc test (q_control-60s_ = 0.003; q_control-90s_ = 0.003, q_15–60s_ = 0.003, q_15–90s_ = 0.006, q_30–60s_ = 0.002, q_30–60s_ = 0.002, *p* < 0.05). In case of ISO 35.06, a similar trend may be observed (see Figure 1a). However, the ANOVA analysis did not confirm the difference in the average PCA concentration between tested activation at the level of significance α = 0.05. Furthermore, analysis using the *t*-test indicated there is no significant difference between PCA release using ISO 30.06 and ISO 35.06 both for 60 s (*p* = 0.146; t_n=3_ = 1.803) and 90 s (*p* = 0.06; t_n=3_ = 2.593).

### 2.3. PCA Release from Shelf-Stored 2% CHX

**Figure 2:**
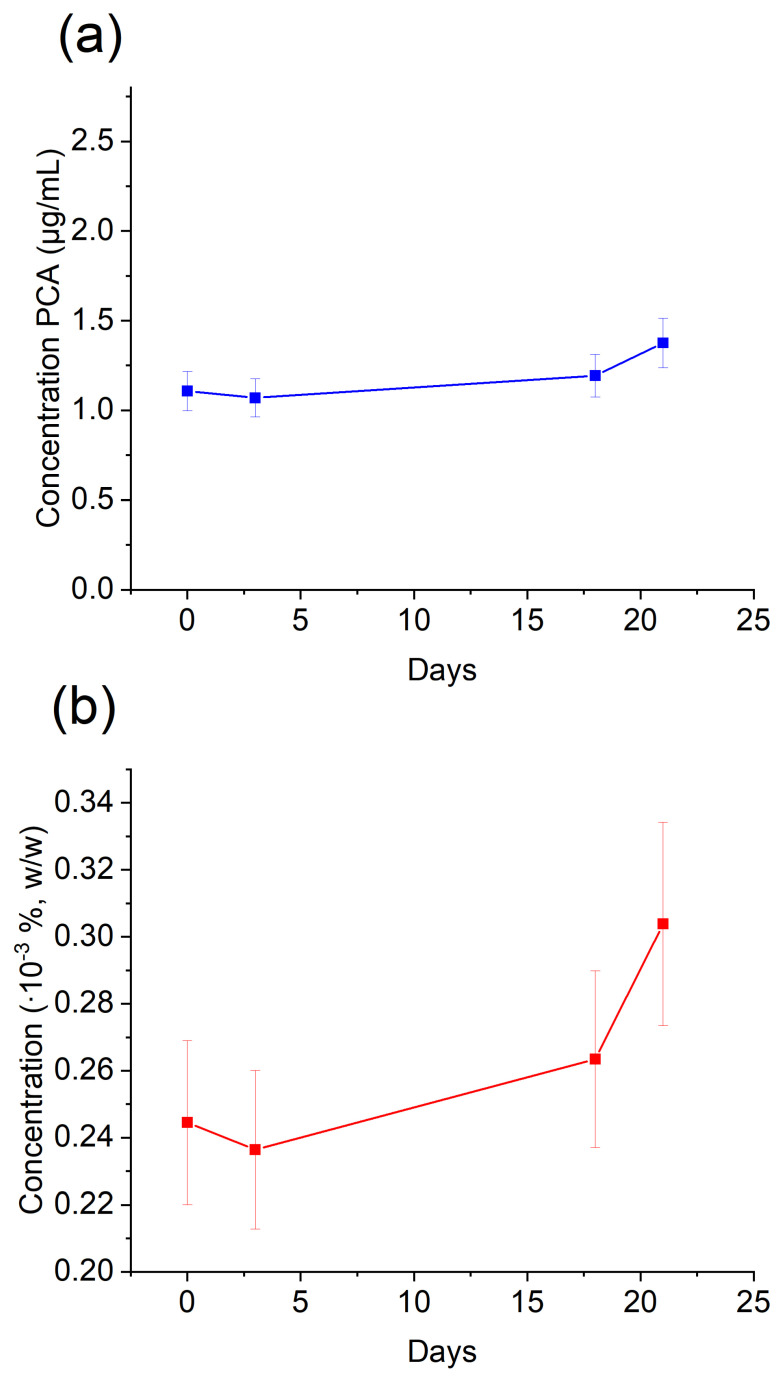
PCA concentration changes found in CHX 2% stored in ambient conditions. (**a**)—data in absolute values; (**b**)—concentration in percentage.

PCA was detected in negative control in samples of 2% CHX collected from shelf-stored 2% CHX and the concentration of PCA was related to the time of storage. The concentrations of PCA were assessed after 0, 3, 18, and 21 days of storage for each experimental group for the volume the bottle of Gluco-CHeX solution (400 g) (Figure 2).

## 3. Discussion

The root canal system can be considered as a closed system, where laminar flow of endodontic solutions should be changed into a turbulent one, in order to enhance cleaning efficacy of root canal irrigants with ultrasonic or manually generated streaming forces. As dentists use IAT more frequently and 2% CHX was proposed to be agitated with those techniques [11,17], it is crucial to assess the safety of this treatment technique in the aspect of PCA formation. PCA is toxic [25], cancerogenic [26], and it can cause methemoglobinemia [27]. While PCA release from the interaction between CHX and other endodontic antiseptics, such as sodium hypochlorite [28] and calcium hydroxide [29], is a well-known fact, its formation during CHX agitation with US was only hypothesized. Furthermore, MDI was thought to be a method of choice for CHX agitation, as it did not heat the solution and, thus, it was postulated that it will not lead to PCA release. However, there was no direct scientific evidence provided regarding whether MDI activation of 2% CHX does not cause PCA formation.

Basrani et al. [21] warmed 2% CHX solution in a water bath for 45 min and detected PCA release at 45 °C but not at 37 °C. After this experiment, US activation of 2% CHX was hypothesized to be dangerous, as PUI increases the temperature of irrigation solutions, but there was no direct proof provided for PCA formation from 2% CHX activated with US. Moreover, a quantitative analysis of PCA release from 2% CHX activated with the US method has not previously been conducted. We confirmed in our experiment that there is PCA release from 2% CHX activated with the US method. For US tip ISO size 30, we observed a rise of PCA concentration over time for all times of activation, while in the ISO 35 group, the PCA amount increased for 15 s, 30 s, and 60 s time of agitation. Moreover, once again, we confirmed the result from previous research, that the PUI method increases the temperature of CHX solutions. However, the temperature rise did not correlate with the agitation time or size of the ultrasonic tips, contrary to previous studies [20,30]. These differences can be explained with the fact that our in vitro experimental model was closer to a clinical treatment methodology, in terms of solution volume and times of activation, as well as the endodontic equipment used. The sizes of the ultrasonic tips (ISO 30 and ISO 35) were chosen as those most widely used for this purpose in clinical practice. The general rule is that the tip should oscillate freely in the root canal without touching the walls in order to produce the most effective acoustic streaming. Smaller sizes of ultrasonic files tend to separate more frequently inside the endodontic space; therefore, ISO 30 and 35 files seems to be the sizes of choice with reasonable consensus. Moreover, the times of activation 15 s and 30 s were chosen as those applied for US agitation in clinics and 60 s and 90 s were inspected for correlation and trend-observing purposes.

Since the MDI method does not cause a temperature rise of 2% CHX, it was hypothesized to be safer and the preferable method of CHX activation in RCT. However, although we confirmed that the MDI method did not cause a temperature rise of 2% CHX solution, we detected PCA in 2% CHX samples after MDI activation. In the MDI method, we used bigger taper gutta-percha cones (35 ISO size 6% taper and 30 ISO size 6% taper), as it was proved that tapered cones are more effective in this technique than non-tapered ones [18]. When a bigger size GP cone was used in the MDI method (35.06), we observed initial PCA decrease when comparing to non-agitated solution, with PCA rise after 60 and 90 s of agitation. This can be hypothetically explained with PCA sedimentation on GP cones, and the total mass and volume of 35.06 cone is bigger than 30.06, which can explain why in 30.06 MDI samples, this effect was not observed. This hypothesis requires further investigation.

Moreover, we detected PCA in the control samples, as well as in the bottle of shelf-stored 2% CHX, which once again confirmed self-degradation of CHX solutions during shelf storage over time [29,31]. This finding shows that it would be preferable to use a high volume of fresh portions of 2% CHX in irrigation protocol from smaller bottles, in order to finish them quicker and not to over-store them in clinics. Moreover, it emphasizes the importance of proper storage of 2% CHX irrigating solutions, and the future perspective is to inspect how storage conditions (e.g., not proper closure of the bottle, UV light, temperature of storage) influence the quantity of PCA release from 2% CHX solutions.

It is important to know that root canal dentin can be considered as a biological substrate, which through irrigation protocol is prepared for further stage of endodontic treatment, that is, obturation with gutta-percha and sealer application. Sealer penetration into the dentinal tubules is a consequence of effective smear layer removal and can be compromised by PCA formation. Therefore, detection of the formation of reactive compounds during chemical preparation of root canals is important in the aspect of obtaining proper bonding strength between different types of sealers and dentin [32,33]. This problem requires further research.

## 4. Materials and Methods

The solid 4-chloroaniline (PCA) standard (Sigma-Aldrich, St. Louis, MO, USA, purity 99%) was dissolved in methanol to obtain a of 10 mg/mL stock solution and then it was stored at −20 °C. Gluco-Chex (Cerkamed, Poland) containing 2% Chlorhexidine digluconate (CHX)—a solution used for root canal irrigation—was used in all experiments. The solution was stored in conditions advocated by the manufacturer.

Acetate buffer of pH 6.0 and ionic strength 150 mmol/L was prepared by adding 160 μL of 2 mol/L acetic acid (Sigma-Aldrich, USA, purity 99.8%) and 7.5 mL of previously prepared 1 mol/L ammonium acetate to a 50 mL volumetric flask, then filled up to line with ultrapure water. Then, 1 mol/L ammonium acetate was prepared by dissolving ca. 1.927 g of solid ammonium acetate (Sigma-Aldrich, USA) in 25 mL of ultrapure water.

Ultrapure water (18.2 MΩcm, TOC < 5 ppb) was obtained using the Milli-Q Plus system (Merck-Millipore, Burlington, MA, USA). Other reagents used, among others, as mobile phase and for syringe rinsing in the liquid chromatograph were methanol (Honeywell, purity for LC-MS ≥ 99.9%) and acetonitrile (Honeywell, purity for LC-MS ≥ 99.9%). Dichloromethane (Sigma-Aldrich) was used for extraction of a target compound.

### 4.1. Instrumentation

The LaChrom (Merck-Hitachi, Tokyo, Japan) high-performance liquid chromatograph coupled with photodiode array detector (detection at 210 nm) was used during all experiments. It was equipped with a Spheri-5 C_18_ separation column (100 × 4.6 mm, 5 µm, Perkin Elmer, Waltham, MA, USA). The separation was carried out in gradient mode at 40 °C using the flow rate of 1 mL/min. Ultrapure water and acetonitrile were used as phase A and phase B, correspondingly. The gradient program started with 50% of phase A, which was kept for 7 min, A decreased down to 5% within 2 min, then kept for the following 4 min. The mobile phase composition was then restored within 2 min to initial conditions and the column was equilibrated for 4 min. Total time of analysis was 20 min. Blank samples were injected each time after the analysis of a sample to rinse the chromatographic system and, thus, avoid the carryover effect. The sample injection volume was 15 μL.

### 4.2. Design of Experiment: Experimental Groups and CHX Agitation Procedure with Dental Equipment

**Figure 3:**
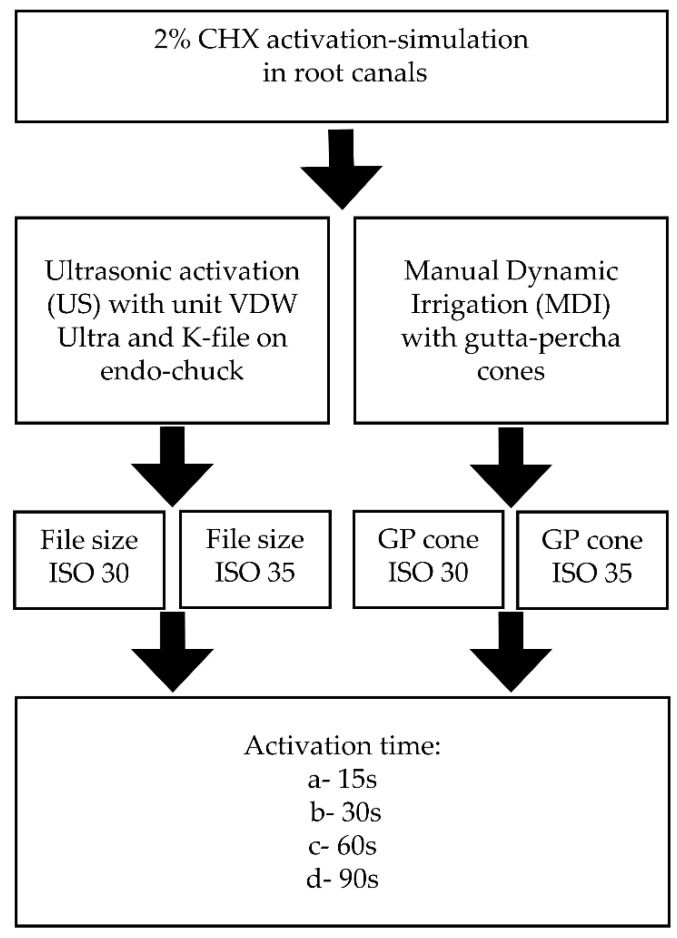
Experimental groups scheme.

To investigate various conditions affecting the stability of CHX during endodontic treatment, two experimental groups were designed depending on the type of activation procedure using 2% CHX solution: ultrasonic (US) and manual dynamic irrigation (MDI). The general scheme of the experiment is depicted in Figure 3.

In US and MDI, the amount of 150 µL of 2% CHX in 200 µL PCR probe was activated for four different times of activation: 15, 30, 60, and 90 s, and each experiment was done in triplicate. Additionally, US and MDI activation techniques were divided into two subgroups depending on tip or GP cone size: for US agitation file size 30 and 35 ISO were used and in MDI group the same sizes of gutta-percha cones were applied. This was set in order to mimic the conditions that occur in the endodontic root canal space. US activation of CHX was done with Endodontic Ultrasonic unit (VDW, Frankfurt am Main, Germany) with dedicated ultrasonic K-File mounted on endo-chuck tip in two sizes: ISO 30 and ISO 35 (size 0.3 mm in diameter at the tip and 0.35 mm in diameter at the tip, respectively, both manufactured by VDW, Germany) mounted on 90° endo-chuck with an oscillation frequency of 28–36 kHz. The US file, when introduced into the probe, oscillated freely inside it with the intention of not touching its walls.

The MDI agitation method of 2% CHX was done with ISO 30 GP cone (size 0.3 mm in diameter at the tip) and ISO 35 GP cone (size 0.35 mm in diameter at the tip). Six percent taper GP cones were used, which means that the diameter of the cone increased with 0.06 mm for every mm of its length. The frequency of push-pull motion of the GP cone was ~3.3 Hz (~100 strokes per 30 s).

Changes in the temperature of the CHX solutions were inspected in all samples for US and MDI groups before and after every activation.

### 4.3. Preparation of Samples for HPLC Analysis

The pre-activated samples were mixed for a few seconds on a shaker to homogenize solutions. Subsequently, 150 μL of the sample was transferred to a fresh 1.5 mL Eppendorf-type tube and 300 μL of acetate buffer, pH 6.0, was added. The pH of the buffer was chosen so that p-chloroaniline was present in the solution in a deprotonated (hydrophobic) form, while CHX was protonated and prone to stay in a water-based phase. In a further step, the PCA was extracted three times by adding 0.5 mL of dichloromethane to the samples each time. After the addition of each portion, the samples were vortexed for 5 min at 2100 rpm and then centrifuged for 5 min (11,200× *g*). The separated DCM layer (0.4 mL) was transferred to a new 1.5 mL Eppendorf-type tube. The collected extracts were vortexed for a few seconds and then 1.2 mL of extract was transferred to a new 1.5 mL Eppendorf-type tube and evaporated under the gentle stream of nitrogen at room temperature (~23 °C). To the resulting precipitate, 50 μL of ACN:H_2_O solution (20:80, *v*/*v*) was added and vortexed for 5 min (2100 rpm). Once the precipitate was dissolved, 20 μL of the sample was transferred to an amber glass vial with a 100 μL microinsert, diluted with 80 μL with ACN:H_2_O solution (20:80) and subjected to the chromatographic analysis.

### 4.4. Statistical Analysis

Data acquired during the study were processed using MS Excel (Office 365, Microsoft, Redmond, WA, USA) for data collection, pretreatment, and calculations. The figures were prepared using Origin 2020 (OriginLab, Northampton, MA, USA) software.

The statistical analysis was accomplished in Statistica 13.3 PL (Tibco, Palo Alto, CA, USA). The results were compared using the one-way analysis of variance (ANOVA), taking the level of significance α = 0.05. Initially, data were tested for equality of the variance using the Levene test. Once the difference between means was detected, the post-hoc analysis with the Tukey test was performed. In cases when the assumptions of the analysis of variance were not met, particularly when the Levene test indicated a significant difference in variance, the Kruskal–Wallis test was applied at the level of significance α = 0.05, followed by the Dunn post-hoc test.

## 5. Conclusions

In conclusion, we would like to come back to the null hypotheses put at the beginning of the study. The mean concentrations of PCA released during activation were not higher than those observed from the self-degradation of shelf-stored 2% CHX. When 2% CHX was activated with US, PCA release was not dependent on the size of the ultrasonic file but was dependent on the time of activation (*p* < 0.05). In the MDI method, a similar trend was observed visually, nevertheless without statistical significance (*p* > 0.05). We did not observe a significant difference between PCA release using ISO 30.06 and ISO 35.06 GP cones (*p* > 0.05) when 2% CHX was activated with the MDI method. On the basis of these findings, it can be concluded that PCA is released during CHX activation with both US and MDI methods. However, these values are still lower than the threshold given in approved norms [34]. This suggests that PCA release from 2% CHX agitated with irrigation agitation techniques may not pose a higher safety risk in the root canal final irrigation protocol, compared to the degradation of CHX over time when stored on the shelf. Therefore, at this state of research, on the activation of 2% CHX with US and MDI methods we did not find increased generation of p-chloroaniline, which is considered a major chemical hazard related to the agitation of 2% CHX in root canal treatment. However, dental practitioners should undertake precautions when using CHX in the endodontic treatment. These clinical considerations include the use of fresh portions of 2% CHX and short time of storage of CHX bottles, as well as not prolonging the time of activation with agitation techniques in the endodontic irrigation protocol, in order to ensure patient safety and optimal treatment outcomes.

The conducted research provides valuable insights into the stability of 2% chlorhexidine (CHX) under various storage conditions, opening up avenues for future investigations. This will facilitate the improvement of consumable management in endodontic practice, allowing for enhanced quality control and product optimization.

It is important to note that while the study provides valuable insight into PCA release as a result of CHX activation, further research and evaluations may be needed to comprehensively assess the safety and potential implications of PCA formation in the endodontic treatment and patient general health condition. The experiments presented in this study were limited to in vitro settings, which provide a preliminary understanding of the degradation process. However, to fully assess the safety and efficacy of root canal irrigation with 2% CHX, it is essential to further investigate potential para-chloroaniline (PCA) deposits within artificial root canals, such as those created using 3D printing techniques, in order to inspect its potential to create microleakage between the root canal wall and obturating material and, therefore, compromise the quality of root canal obturation. Additionally, expanding the research to include animal experiments would provide a more realistic evaluation of the safety and potential adverse effects of the irrigation procedure in order to examine if released amounts of PCA have any influence on patient general health condition [25,27].

Furthermore, it is worthwhile to explore other medicaments, such as mouth rinsing liquids containing CHX, as potential sources of p-chloroaniline. This broader evaluation would contribute to a comprehensive understanding of the potential formation and exposure routes of PCA in clinical settings.

## Figures and Tables

**Table 1:** Changes of 2% CHX solution before and after agitation with US and MDI.

Activation Type	Activation Time [s]	Temperature Change ΔT, [°C]	
		ISO 30	ISO 35
US	15	+3	+4
	30	+3	+3
	60	+3	+3
	90	+4	+4
		ISO 30	ISO 35
MDI	15	0	0
	30	0	0
	60	0	0
	90	0	0

## Data Availability

Not applicable.

## Sample Availability

Samples of the compounds are not available from the authors.
